# A Comparative Study of Clinical Outcomes Between Cruciate-Retaining and Posterior-Stabilized Total Knee Arthroplasty: A Propensity Score-Matched Cohort Study

**DOI:** 10.7759/cureus.45775

**Published:** 2023-09-22

**Authors:** Keiichiro Yamamoto, Arata Nakajima, Masato Sonobe, Yorikazu Akatsu, Manabu Yamada, Koichi Nakagawa

**Affiliations:** 1 Orthopaedic Surgery, Toho University Graduate School of Medicine, Tokyo, JPN; 2 Orthopaedic Surgery, Toho University Sakura Medical Center, Sakura, JPN

**Keywords:** clinical outcomes, posterior stabilized, cruciate retaining, propensity score matching, total knee arthroplasty

## Abstract

Introduction: We investigated a comparison of clinical outcomes between cruciate-retaining (CR) and posterior-stabilized (PS) total knee arthroplasty. However, it is still controversial which design leads to better clinical results. In clinical settings, choosing either CR or PS is likely based on the surgeon’s preferences. In this study, short-term clinical outcomes between CR and PS in patients who received a single knee prosthesis were compared using propensity score matching.

Methods: Two hundred and twelve CR and 43 PS of a single knee prosthesis were enrolled in this study. After propensity score matching, 34 knees each in the CR and PS groups were chosen and were without significant differences in age at operation, gender, BMI, preoperative range of motion (ROM), preoperative femorotibial angle (FTA), and presence or absence of patellar replacement. Clinical scores, including ROM, Knee Society score (KSS), knee injury and osteoarthritis outcome score (KOOS), except for the sports subscale, were compared between the CR and PS groups preoperatively and two years postoperatively.

Results: Postoperatively, there were no significant differences in FTA, ROM, or KSS. Preoperative scores for the KOOS except for the pain subscale were comparable between the groups. Postoperatively, however, the PS group had a significantly higher score in the ADL subscale compared to the CR group (PS: 89.5 vs. CR: 80.8, p = 0.017). The KOOS subscales other than activities of daily living (ADL) were comparable between the groups.

Conclusions: In this propensity score-matched cohort study, PS showed a better outcome for the ADL than the CR design. These findings suggest that choosing either CR or PS should not depend on the surgeon’s preferences. A PS design may be preferable to CR for elderly patients.

## Introduction

Many orthopedic surgeons have investigated the comparison of clinical outcomes between cruciate-retaining (CR) and posterior-stabilized (PS) total knee arthroplasty (TKA) [1-8]. However, it is still controversial and leads to better clinical results. In CR TKA, the posterior cruciate ligament (PCL) and inter-condyle of the femur are preserved. Therefore, CR is likely to be chosen for patients with relatively younger ages or less severe deformities. Cruciate-retaining is also supposed to function better in terms of proprioception, balance, and kinematics [9]. In PS TKA, the PCL and inter-condyle of the femur are resected; hence, PS is likely to be chosen for patients with older age or severe deformities and is supposed to lead to higher degrees of flexion and better femoral roll-back [10]. A recent meta-analysis on the comparison of clinical outcomes between CR and PS revealed no differences in knee scores, range of motion, or complication rates [11]. A possible reason for no difference in clinical results between CR and PS is considered to be the use of multiple kinds of prostheses and the recruitment of patients with different preoperative characteristics.

The ideal method to compare clinical outcomes between CR and PS is considered to be a randomized controlled trial. However, it is not realistic to randomly assign patients to different types of prostheses due to ethical reasons. Propensity score matching is a statistical method to analyze differences between two groups with different characteristics at baseline [12]. This method allows us to know significant differences in the CR and PS, even in patients with different preoperative characteristics.

This study aims to compare short-term clinical outcomes between CR and PS in patients who received a single knee prosthesis and whose cohorts were matched by preoperative characteristics. We hypothesized that knee scores and patient-reported outcomes would be comparable between CR and PS.

## Materials and methods

Recruitment of patients

**Figure 1:**
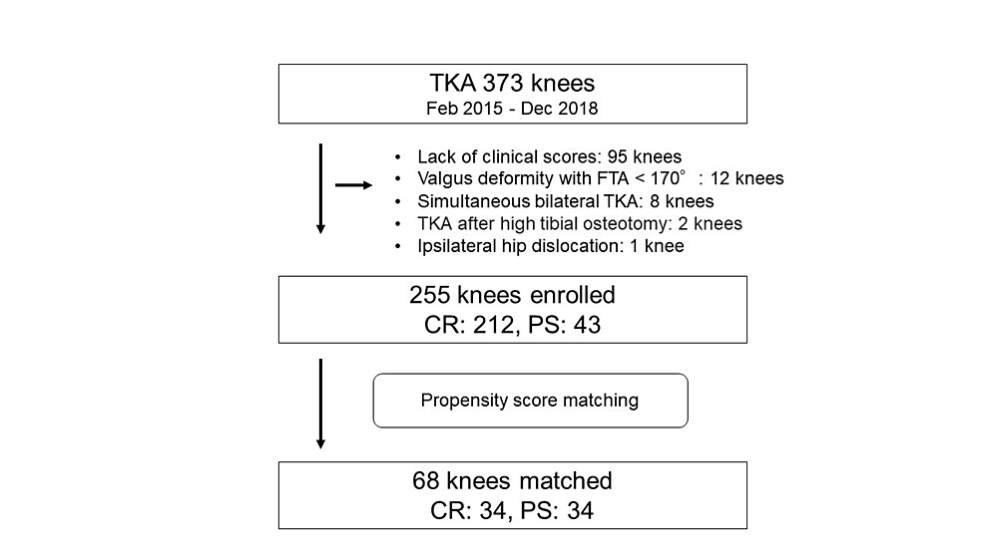
Patient flow diagram CR: Cruciate-retaining, PS: Posterior-stabilized, FTA: Femorotibial angle, TKA: Total knee arthroplasty

This study retrospectively recruited 373 knees that had undergone TKA with a single prosthesis (FINE Knee, Teijin-Nakashima Medical Co. Ltd., Okayama, Japan) at our hospital between February 2015 and December 2018. It was approved by the Ethics Committee of Toho University Sakura Medical Center (approval number: S23005). All patients received either the CR or PS type of the FINE knee. A total of 118 knees were excluded due to the following reasons: lack of clinical scores (95 knees), valgus deformity with femorotibial angle (FTA) < 170° (12 knees), simultaneous bilateral TKA (8 knees), TKA after high tibial osteotomy (2 knees), ipsilateral hip dislocation (1 knee). As a result, 212 knees from the CR group and 43 knees from the PS group were enrolled in this study. In this study period, we preferentially used CR if the PCL was intact on the MR images, while PS was used only if the PCL was severely degenerated or deficient. A total of 255 knees were subjected to propensity score matching (Figure 1).

Surgical procedures

All TKAs were performed using the measured resection technique through the mid-vastus approach. Our goal was mechanical neutral alignment, and surgeries were performed using conventional instruments. We decided prior to surgery which type of implant (CR or PS) we would use. Basically, we chose CR if the PCL was intact on the MR images, but PS in cases of severe flexion contracture and/or severe varus deformity. A distal femoral osteotomy was conducted perpendicular to the mechanical axis at a level of 9 mm to 10 mm from the farthest point of the medial condyle, and the posterior condyle was osteotomized parallel to the surgical epicondylar axis. A tibial osteotomy was subsequently conducted perpendicular to the anatomical axis of the tibia. The resection level was 8 mm to 10 mm distal to the convexity of the lateral plateau. Following osteotomy, soft tissue balance was checked both in extension and flexion using the knee balancer. When the minimum gap to implant the prosthesis was not obtained on the medial side, the MCL was released from the tibial site, or the pie-crust release was added. After no flexion contracture and a good flexion angle in replacement with trial components were confirmed, the implants were fixed to the bone with cement. We replaced patellae, but not for patients without osteoarthritic changes in the patellofemoral joint or with small patellae. Patients were discharged three weeks after surgery when they were medically stable, with pain controlled by oral analgesics, and when deemed by a physiotherapist to be mobilizing sufficiently to function safely at home.

Clinical evaluation

Clinical scores, including range of motion (ROM), Knee Society score (KSS), knee score (KS), function score (FS) [13], and knee injury and osteoarthritis outcome score (KOOS) [14], were taken into account except for the sports subscale, which was recorded preoperatively and two years postoperatively. The ROM was measured by a single observer (K.Y.) using a goniometer with patients laid on a flat table. The ROM was calculated by subtracting the maximum extension angle from the maximum flexion angle. Changes in the scores between preoperative and postoperative (Δscores) were also recorded.

Propensity score matching and statistical analysis

Propensity score matching was performed between 212 knees from the CR group and 43 knees from the PS TKA group. When calculating the propensity score by multivariate logistic regression analysis in each patient, the following variables were included: age at operation, gender, BMI, preoperative ROM, preoperative standing FTA, and the presence or absence of patellar replacement. Consequently, the 34 patients were matched for the propensity score-matched analysis in each group (as seen in Figure 1). The Mann-Whitney U-test and the chi-squared test were used to analyze significant differences between groups.

All statistical analyses were performed using the EZR software (Saitama Medical Center Jichii Medical University, Saitama, Japan), which is a modified version of R Commander designed to add statistical functions frequently used in biostatistics [15]. Results were expressed as the mean±SD, and p-values < 0.05 were considered statistically significant.

## Results

Demographic data of the patients after propensity score matching

After propensity score matching, each of the 34 knees in the CR and PS groups was chosen. All matched preoperative valuables, including age at operation, gender, BMI, ROM, FTA, and presence or absence of patellar replacement, were identical, with no significant differences between the CR and PS groups (Table 1).

**Table 1:** Preoperative demographic data of the patients after propensity score matching CR: Cruciate-retaining, PS: Posterior-stabilized, ROM: Range of motion, FTA: Femorotibial angle

Characteristics	CR	PS	p-value	Standardized difference
Number of patients	34	34		
Age (years)	76.2 ± 6.9	76.0 ± 6.6	0.886	0.035
Gender (male/female)	7/27	5/29	0.752	0.155
BMI	26.7 ± 4.6	26.8 ± 5.9	0.619	0.027
ROM (°)	96.9 ± 23.4	99.0 ± 21.8	0.777	0.091
FTA (°)	186.9 ± 5.4	187.5 ± 6.1	0.708	0.107
Patellar replacement	18 (52.9%)	22 (64.7%)	0.460	0.241

Comparison of clinical outcomes between the CR and PS groups

Preoperative ROM and KSS (KS and FS) in both groups are shown in Table 2. There were no significant differences in the KS and FS between groups. Postoperatively, there were no significant differences in FTA, ROM, KS, or FS. Changes in ROM, KS, and FS (Δ) were comparable between the CR and PS groups.

**Table 2:** Comparison of FTA, ROM, and KSS between CR and PS groups CR: Cruciate-retaining, PS: Posterior-stabilized, ROM: Range of motion, FTA: Femorotibial angle, KS: Knee score, FS: Function score

Subscales	CR (n = 34)	PS (n = 34)	p-value
Postoperative FTA (°)	175.6 ± 3.1	176.2 ± 2.1	0.257
Preoperative ROM (°)	96.9 ± 23.4	99.0 ± 21.8	0.777
Postoperative ROM (°)	118.4 ± 15.0	122.2 ± 10.2	0.443
ΔROM (°)	21.5 ± 22.0	23.2 ± 17.9	0.502
Preoperative KS	37.1 ± 14.6	37.9 ± 14.8	0.830
Postoperative KS	96.4 ± 5.5	97.4 ± 3.4	0.869
ΔKS	59.3 ± 15.1	59.5 ± 14.6	0.971
Preoperative FS	33.8 ± 19.3	33.5 ± 20.0	0.975
Postoperative FS	64.6 ± 22.5	69.6 ± 17.6	0.377
ΔFS	30.7 ± 25.2	36.0 ± 18.2	0.213

Preoperative scores for the KOOS except for the pain subscale were comparable between groups. Postoperatively, however, the PS group had a significantly higher score in the activities of daily living (ADL) subscale compared to the CR group (PS: 89.5 vs. CR: 80.8, p = 0.017) (Table 3). The KOOS subscales other than ADL were comparable between groups. There were no significant differences in changes in all the KOOS subscales (Δ) between the CR and PS groups.

**Table 3:** Comparison of KOOS subscales between the CR and PS groups KOOS: Knee injury and osteoarthritis outcome score, CR: Cruciate-retaining, PS: Posterior-stabilized, ADL: Activities of daily living, QoL: Quality of life

KOOS	CR (n = 34)	PS (n = 34)	p-value
Preoperative symptom	47.7 ± 21.4	49.3 ± 22.6	0.593
Postoperative symptom	84.7 ± 11.8	87.3 ± 11.6	0.348
ΔSymptom	37.0 ± 21.7	38.0 ± 23.5	0.672
Preoperative pain	34.2 ± 20.3	45.7 ± 19.9	0.028*
Postoperative pain	88.6 ± 13.4	91.6 ± 8.5	0.683
ΔPain	54.4 ± 20.7	45.9 ± 22.6	0.088
Preoperative ADL	56.4 ± 17.0	57.5 ± 16.9	0.854
Postoperative ADL	80.8 ± 15.6	89.5 ± 7.9	0.017*
ΔADL	24.4 ± 17.6	32.0 ± 17.8	0.088
Preoperative QOL	21.5 ± 11.8	23.7 ± 16.8	0.834
Postoperative QOL	66.0 ± 23.1	74.4 ± 14.9	0.160
ΔQOL	44.4 ± 26.5	50.7 ± 21.7	0.302

## Discussion

Over the past decades, there have been several systematic reviews and meta-analyses investigating differences in clinical outcomes between CR and PS TKA [1,16]. However, there is no conclusion as to whether one type is superior to the other. This may be due to a lack of randomized controlled trials evaluating clinical results between CR and PS using a single knee prosthesis. Meanwhile, it has been shown that the preoperative clinical and radiological evaluation of the patient has a predictive value in determining the outcome, whether it is CR or PS [16]. In clinical settings, choosing either CR or PS is likely based on the surgeon’s preference and the existing pathology of the PCL [17]. A systematic review analyzing a total of 5407 conventional TKA showed comparable functional improvement between CR and PS [16]. Furthermore, a recent meta-analysis demonstrated an increase in ROM in conventional PS implants compared to CR [18]. Traditionally, PS has been reported to achieve a greater ROM compared to CR [19] because PS design can prevent the paradoxical anterior translation during flexion that is often seen in CR TKA and may decrease the flexion angle [20]. In the present study, however, there was no significant difference in postoperative ROM between CR and PS, and both types achieved a significant increase in flexion angle postoperatively. These observations suggest that paradoxical anterior translation may be inhibited in the CR as well as in the PS design of the FINE Knee that was used in this study. The asymmetric condylar design of the FINE Knee also allows for posterior condylar offset, which helps retain the isometry of the MCL during motion. Appropriate tension of both MCL and PCL during motion may contribute to inhibition of the paradoxical anterior translation.

There have been few previous studies investigating clinical outcomes comparing CR with PS design using matched pair analysis or propensity score matching [21,22]. Thus far, there is only one report that compares clinical results between CR and PS in a matched-pair cohort [21]. The authors state that both groups had similar postoperative clinical scores, including KSS, Oxford knee score, and Short Form-36 Health Survey. In this study by Soong et al., however, two kinds of knee prostheses with different design concepts were used; therefore, an exact comparison of clinical results between CR and PS is impossible. In Soong et al.'s report, the mean age at operation was 70 years old, which seems younger than the population that we included in the present study (76 years old). To the best of our knowledge, our present study is the first report comparing clinical outcomes between CR and PS of a single knee prosthesis using a propensity score-matched cohort.

In our present study, after propensity score matching, the age matched at operation was 76 years old in both groups. So, are PCLs functioning normally in such elderly patients? Kleinbart et al. performed a histological comparison of PCL from arthritic and age-matched knee specimens and found that only 17% of the arthritic group was normal [23]. Similar results were reported by Mullaji et al., who found that PCLs were moderately degenerated in most knees, irrespective of the grade of arthritis and severity of the deformity [24]. These findings suggest that PCL is degenerated moderately to severely in most osteoarthritic knees and may not function normally if it is retained in CR TKA. However, this is not to say that a CR design should be avoided for elderly patients because many studies have reported successful TKAs that retain a PCL [25-29]. A possible explanation for our observation that the ADL subscale in the PS group was superior to the CR group is that there might be patients whose PCL was severely degenerated and did not function normally in patients receiving the CR implants, considering that the mean age at operation was 76 years old.

Regarding how much of the degenerated PCL can be retained in TKA, it is difficult to know PCL function on preoperative MRI alone. A few papers were available on predictors of PCL degeneration in osteoarthritic knees [30]. Aggarwal et al.'s study used advanced osteoarthritic knees receiving TKA, and the authors stated that preoperative KSS, anteroposterior instability, anterior cruciate ligament (ACL) appearance and insufficiency, and erosion in the lateral tibiofemoral compartment were predictors of PCL degeneration. They also commented that ACL appearance, which was graded as normal, abnormal, or ruptured, correlated positively with PCL degeneration, but PCL degeneration did not correlate with flexion deformity or varus deformity. Taken together with these findings, intraoperative ACL appearance can help the surgeon’s decision on the optimal use of a CR or PS design.

There are some limitations to this study. First, the sample size is relatively small, and the patients were recruited from a single institution. To compare clinical outcomes between CR and PS, more cases will be required from multiple institutions. This study was a propensity-matched cohort study, but a prospective randomized controlled trial would also be desirable. Second, three surgeons (AN, YA, and KN) were involved in performing TKA; however, they operated using the same surgical technique (neutral alignment strategy, mid-vastus approach, and measured resection). The decision to replace the patellar or not was dependent on different surgeons. Although the presence or absence of patellar replacement was adjusted between the groups after propensity score matching, it could not be ignored when investigating the influence of implant design (CR/PS) on postoperative outcomes. Third, this study was a comparison of short-term clinical results; therefore, a long-term follow-up will be required to compare clinical outcomes between the CR and PS designs.

## Conclusions

The short-term clinical outcomes between CR and PS TKA using a propensity score-matched cohort were comparable. However, the ADL subscale of the KOOS was significantly better in the PS group than in the CR group. In TKA, choosing either CR or PS should not depend on the surgeon’s preferences. Also, a PS design may be preferable to CR for elderly patients.

## References

[1] Li C, Dong M, Yang D, Zhang Z, Shi J, Zhao R, Wei X (2022). Comparison of posterior cruciate retention and substitution in total knee arthroplasty during gait: a systematic review and meta-analysis. J Orthop Surg Res.

[2] Dalton P, Holder C, Rainbird S, Lewis PL (2022). Survivorship comparisons of ultracongruent, cruciate-retaining and posterior-stabilized tibial inserts using a single knee system design: results from the Australian Orthopedic Association National Joint Replacement Registry. J Arthroplasty.

[3] Broberg JS, Ndoja S, MacDonald SJ, Lanting BA, Teeter MG (2020). Comparison of contact kinematics in posterior-stabilized and cruciate-retaining total knee arthroplasty at long-term follow-up. J Arthroplasty.

[4] Bontempi M, di Sarsina RT, Muccioli MGM, Pizza N, Cardinale U, Bragonzoni L, Zaffagnini S (2020). J-curve design total knee arthroplasty: the posterior stabilized shows wider medial pivot compared to the cruciate retaining during chair raising. Knee Surg Sports Traumatol Arthrosc.

[5] Seon JK, Park JK, Shin YJ, Seo HY, Lee KB, Song EK (2011). Comparisons of kinematics and range of motion in high-flexion total knee arthroplasty: cruciate retaining vs. substituting designs. Knee Surg Sports Traumatol Arthrosc.

[6] Arbuthnot JE, Wainwright O, Stables G, Rathinam M, Rowley DI, McNicholas MJ (2011). Dysfunction of the posterior cruciate ligament in total knee arthroplasty. Knee Surg Sports Traumatol Arthrosc.

[7] Harato K, Bourne RB, Victor J, Snyder M, Hart J, Ries MD (2008). Midterm comparison of posterior cruciate-retaining versus -substituting total knee arthroplasty using the Genesis II prosthesis. A multicenter prospective randomized clinical trial. Knee.

[8] Swanik CB, Lephart SM, Rubash HE (2004). Proprioception, kinesthesia, and balance after total knee arthroplasty with cruciate-retaining and posterior stabilized prostheses. J Bone Joint Surg Am.

[9] Conditt MA, Noble PC, Bertolusso R, Woody J, Parsley BS (2004). The PCL significantly affects the functional outcome of total knee arthroplasty. J Arthroplasty.

[10] Jiang C, Liu Z, Wang Y, Bian Y, Feng B, Weng X (2016). Posterior cruciate ligament retention versus posterior stabilization for total knee arthroplasty: a meta-analysis. PLoS One.

[11] Rosenbaum P, Rubin DB (1983). The central role of the propensity score in observational studies for causal effects. Biometrika.

[12] Insall JN (1989). Rationale of the Knee Society clinical rating system. Clin Orthop.

[13] Roos EM, Roos HP, Lohmander LS, Ekdahl C, Beynnon BD (1998). Knee injury and osteoarthritis outcome score (KOOS)—development of a self-administered outcome measure. J Orthop Sports Phys Ther.

[14] Kanda Y (2013). Investigation of the freely available easy-to-use software 'EZR' for medical statistics. Bone Marrow Transplant.

[15] Longo UG, Ciuffreda M, Mannering N, D'Andrea V, Locher J, Salvatore G, Denaro V (2018). Outcomes of posterior-stabilized compared with cruciate-retaining total knee arthroplasty. J Knee Surg.

[16] Kaya O, Pihtili Tas N, Batur OC, Gonder N (2023). Correlation of radiological and functional results while determining total knee prosthesıs surgery indication in patients with osteoarthritis. Firat Med J.

[17] Kolisek FR, McGrath MS, Marker DR, Jessup N, Seyler TM, Mont MA, Barnes LC (2009). Posterior-stabilized versus posterior cruciate ligament-retaining total knee arthroplasty. Iowa Orthop J.

[18] Migliorini F, Eschweiler J, Tingart M, Rath B (2019). Posterior-stabilized versus cruciate-retained implants for total knee arthroplasty: a meta-analysis of clinical trials. Eur J Orthop Surg Traumatol.

[19] Bercik MJ, Joshi A, Parvizi J (2013). Posterior cruciate-retaining versus posterior-stabilized total knee arthroplasty: a meta-analysis. J Arthroplasty.

[20] Hamai S, Okazaki K, Shimoto T, Nakahara H, Higaki H, Iwamoto Y (2015). Continuous sagittal radiological evaluation of stair-climbing in cruciate-retaining and posterior-stabilized total knee arthroplasties using image-matching techniques. J Arthroplasty.

[21] Soong J, Ou Yang Y, Ling ZM, Chia SL, Lo NN, Yeo SJ (2021). Cruciate retaining and posterior stabilized total knee arthroplasty in severe varus osteoarthritis knee: A match-pair comparative study in an Asian population. J Orthop Surg (Hong Kong).

[22] Koh IJ, Chalmers CE, Lin CC, Park SB, McGarry MH, Lee TQ (2021). Posterior stabilized total knee arthroplasty reproduces natural joint laxity compared to normal in kinematically aligned total knee arthroplasty: a matched pair cadaveric study. Arch Orthop Trauma Surg.

[23] Kleinbart FA, Bryk E, Evangelista J, Scott WN, Vigorita VJ (1996). Histologic comparison of posterior cruciate ligaments from arthritic and age-matched knee specimens. J Arthroplasty.

[24] Mullaji AB, Marawar SV, Simha M, Jindal G (2008). Cruciate ligaments in arthritic knees: a histologic study with radiologic correlation. J Arthroplasty.

[25] Yamada M, Nakajima A, Sonobe M (2023). The impact of postoperative inclination of the joint line on clinical outcomes in total knee arthroplasty using a prosthesis with anatomical geometry. Sci Rep.

[26] Nakajima A, Yamada M, Sonobe M (2021). Three-year clinical and radiological results of a cruciate-retaining type of the knee prosthesis with anatomical geometry developed in Japan. BMC Musculoskelet Disord.

[27] Nakamura J, Inoue T, Suguro T (2018). A comparative study of flat surface design and medial pivot design in posterior cruciate-retaining total knee arthroplasty: a matched pair cohort study of two years. BMC Musculoskelet Disord.

[28] Chun KC, Lee SH, Baik JS, Kook SH, Han JK, Chun CH (2017). Clinical and radiological results of cruciate-retaining total knee arthroplasty with the NexGen®-CR system: comparison of patellar resurfacing versus retention with more than 14 years of follow-up. J Orthop Surg Res.

[29] Sumino T, Rubash HE, Li G (2013). Does cruciate-retaining total knee arthroplasty enhance knee flexion in Western and East Asian patient populations? A meta-analysis. Knee.

[30] Aggarwal AK, Goel A, Radotra BD (2013). Predictors of posterior cruciate ligament degeneration in osteoarthritic knees. J Orthop Surg (Hong Kong).

